# Reply to Hockenhull et al. Comment on “Ssuna et al. Animal Welfare Guidelines for International Development Organisations in the Global South. *Animals* 2024, *14*, 2012”

**DOI:** 10.3390/ani14233447

**Published:** 2024-11-28

**Authors:** Paul Ssuna, Karin Siegmund, Andrew Crump

**Affiliations:** 1 School of Veterinary Medicine and Animal Resources, Animal Welfare Competence Center for Africa (AWeCCA), Makerere University, Kampala 7062, Uganda; 2 Welttierschutzstiftung, 10117 Berlin, Germany; ks@welttierschutz.org; 3 Animal Welfare Science and Ethics, Royal Veterinary College, London NW1 0TU, UK; acrump@rvc.ac.uk

We thank Hockenhull et al., (2024) [1] for their valuable comment on our animal welfare guidelines for international development organisations in the Global South [2]. The commentators raise the issue of working animals, which they expansively define as “any animal that is kept by humans to perform tasks”. In their view, our guidelines made “a significant oversight” through “the omission of reference to the needs and welfare of working animals”. We absolutely agree that working animal welfare is an important issue for international development organisations. Indeed, our guidelines explicitly referenced both working animals and example species, such as donkeys and camelids. We nonetheless appreciate the commentators for further raising awareness of working animal welfare.

Nonetheless, perhaps we should clarify the guidelines’ aim. They were designed to be general, covering all domestic animals used in development projects in the Global South. This includes working animals [3], which were commonly observed on our project visits, but also encompasses animals reared for food or other products, animals kept for breeding or sale, and animals used in scientific research. These are very different contexts, but common welfare needs exist: proper healthcare, nutrition, and housing; well-designed staff training programmes and standard operating procedures; and appropriate transport and euthanasia methods. The guidelines aimed to capture this higher level of thinking about welfare, which applies to all animals in human care. We, thus, believe they do address the “needs and welfare of working animals”, at least in broad terms—just not exclusively. Moreover, too much focus on working animals would have reduced this general applicability; for example, making the guidelines less appropriate to livestock. We nevertheless appreciate this trade-off is ultimately a value judgement, and the commentators’ views and experience are just as valid as our own.

Going forward, we fully support complementing our overarching guidelines with more specific guidelines, which address the welfare needs of particular species or in particular contexts. These would support international development organisations in the Global South with their efforts to improve animal welfare. We would welcome the opportunity to work with the commentators to compile such guidelines for working animals.
